# ‘Boost Camp’, a universal school-based transdiagnostic prevention program targeting adolescent emotion regulation; evaluating the effectiveness by a clustered RCT: a protocol paper

**DOI:** 10.1186/s12889-018-5754-5

**Published:** 2018-07-21

**Authors:** Brenda Volkaert, Laura Wante, Leentje Vervoort, Caroline Braet

**Affiliations:** Department of Developmental, Personality and Social Psychology, Ghent University, Henri Dunantlaan 2, Ghent, Belgium

**Keywords:** Adolescents, Emotion regulation, School-based, Prevention program, Emotional wellbeing

## Abstract

**Background:**

The transition from childhood into adolescence can be considered as a critical developmental period. Moreover, adolescence is associated with a decreased use of adaptive emotion regulation strategies and an increased use of maladaptive emotion regulation strategies increasing the risk of emotional problems. Targeting emotion regulation is therefore seen as an innovative prevention approach. The present study aims to evaluate the effectiveness of Boost camp, an innovative school-based prevention program targeting ER, on adolescents’ emotion regulation skills and emotional wellbeing. Also secondary outcomes and possible moderators will be included.

**Methods:**

The aim is to reach 300 adolescents (16 class groups, 6 schools) in their first year of high school. A clustered Randomized Controlled Trial (RCT) with two conditions, intervention (*n* = 150) and control (n = 150), will be set up. Adolescents in the intervention condition will receive 14 lessons over the course of 2 days, followed by Booster sessions, and will be compared with adolescents in a non-intervention control group. The outcomes will be measured by self-report questionnaires at baseline, immediately after Boost camp, and at three and 6 months follow-up.

**Discussion:**

Data-collection is planned to be completed in May 2018. Data-analyses will be finished the end of 2018. The presented paper describes the Boost camp program and the clustered RCT design to evaluate its effectiveness. It is expected that Boost camp will have beneficial effects. If found effective, Boost camp will have the potential to increase adolescent’s ER and well-being, and reduce the risk to become adults in need. The trials is registered on the 13th of June 2017 in ISRCTN registry [ISRCTN68235634].

## Background

The transition from childhood into adolescence is a developmental period whereby young adolescents have to deal with a wide range of changes and challenges. First of all, this developmental phase is characterized by rapid physical and emotional development, which often coincides with higher levels of negative affect, and higher risk-taking behavior [27, 134]. Second, young adolescents are faced with growing academic expectations, while at the same time they report less school motivation, feel more pressured by schoolwork, and describe decreased perceived school performance [81]. Finally, early adolescence is associated with important social challenges, such as more complex and hierarchical peer relationships, which require refined social skills [135]. Hence, it comes as no surprise that this transition period often co-occurs with heightened levels of stress.

Parallel with the observed changes and challenges, adolescents also report more health complaints, less life satisfaction [81], and a sudden and massive increase in psychopathology symptoms [27]. Approximately 5 to 20% of the adolescents worldwide experience significant impairments due to psychological problems [146, 147]. Syndromes as drug abuse, anxiety, depression, and behavioral disorders show a clear increase in prevalence in early adolescence (i.e., 12 and 15 years) [35]. These findings are of great concern since mental health problems in adolescence are a clear predictor of serious psychopathology in adulthood [44, 146]. Furthermore, young adolescents with psychological problems often fail to find help and the majority of them (50–70%) does not receive (adequate) treatment [33, 34, 96]. It appears that adolescents are faced with various barriers that withhold them from seeking help, such as public, perceived, and self-stigmatizing attitudes to mental illness, general lack of knowledge about mental health service, and poor access to mental health services [24, 68]. The heightened risk for the development of psychological problems, the long-term burden of adolescent psychopathology, and the limited reach of treatment services highlight the importance of prevention and early intervention programs to prevent the development of psychopathology in adolescents [32, 33, 84].

### The role of emotion regulation

Prevention programs should tackle basic mechanisms involved in stress regulation. The transdiagnostic framework suggests that emotion regulation (ER) plays a crucial role in the etiology and maintenance of multiple psychological problems [63]. ER refers to the “processes by which individuals influence *which* emotions they have, *when* they have them and *how* they experience and express these emotions” [65]. In order to manage or modulate the intensity of the emotions experienced, various ER strategies can be used. ER strategies are considered adaptive or maladaptive dependent on their effects on affect and behavior in the long-term and their association with psychopathology [3, 23]. Adaptive ER strategies reduces negative affect and protect against the development of youth psychopathology. Examples are reappraisal, acceptance, distraction, and problem solving [115]. Maladaptive ER strategies are less effective in reducing negative emotion and are related with the development of psychological problems. Examples are rumination, avoidance, and suppression, [3].

Adolescence is known as an important and challenging phase with regard to ER development. First, young adolescents shift from external parent-guided ER strategies, such as support seeking, to internal autonomous ER strategies, such as cognitive reappraisal and problem solving [2, 53, 58]. Second, young adolescents experience more intense negative affect and ER difficulties originating in the different developmental courses between reactive and regulative cognitive brain systems, such as inhibition, working memory, emotional control and planning (defined as executive functions (EF)) [129]. Due to a slower maturation of the executive functions, young adolescents’ brain systems often shows an increased emotional reactivity but a slower activation of regulative cognitive processes when confronted with emotional stimuli [95, 129, 134]. This imbalance is also reflected in a decrease in the use of adaptive ER strategies and an increase in the use of maladaptive ER strategies among adolescents, specifically in 12-to-15-year olds [37, 69, 129, 150]. Both the underuse of adaptive ER strategies and the overuse of maladaptive ER strategies are associated with internalizing symptoms (i.e., depressive and anxiety symptoms) and externalizing symptoms in adolescence (i.e. attention-deficit/hyperactivity disorder and conduct disorder symptoms) [99, 117, 122]. These observations indicates that adolescence is characterized by an increased emotional instability [40, 129, 134], which may explain the increased risk of developing psychopathology in this age group.

Considering that adolescence is characterized by ongoing cognitive and emotional maturation [40, 129, 134], this developmental phase provides great opportunities for therapeutic interventions focusing on emotional wellbeing. Emerging research indicates that programs tackling stress regulation, developed within a transdiagnostic framework and integrating core components of evidence-based interventions (e.g. cognitive reappraisal, modifying action tendencies, prevention of emotional avoidance), have strong potential to improve adolescents’ emotional wellbeing, problem behavior, and academic performance, both in a prevention [36, 49, 51] and clinical context [20, 21, 30, 52, 108, 119, 130].

So far, results of school-based universal social and emotional prevention programs have been mixed, with some showing beneficial effects on competencies and attitudes of children and adolescents, whereas others did not [51]. There are several explanations for this equivocal result. First, adolescents are more eager to learn when programs are tailored to their needs. Consequently, prevention programs may have stronger effects during stressful life changes, such as the critical transition phase from childhood into adolescence or the start of secondary school [51, 55]. Second, up to date, there is no prevention program, exclusively targeting the basic underlying mechanisms related with wellbeing, imbedded in a strong evidence-based framework. Third, the majority of previous research only focuses on the effect on primary outcomes such as social-emotional skills and levels of emotional distress. However, also secondary outcomes are needed to fine-tune effects. For example, a subset of studies have showed positive effects of academic achievement as precursor of increased wellbeing later on [51], influenced by affective and cognitive functioning [70]. Yet, other crucial outcomes might be important to include as well like school drop-out and days absent [79], quality of peer relations [92, 118] and attitudes on mental health [19].

Finally, universal programs do not have the same effects on all participants. Therefore, it is necessary to identify subgroups of youngsters based on crucial variables such as EF, baseline scores on psychopathology, or gender, and explore which subgroups benefit less or more. For example, it is possible that adolescents with low levels of EF benefit less from a prevention program targeting solely ER, given the observations that skilled EF facilitates the learning of ER skills [28]. Furthermore, adolescents with psychological problems report greater declines in mental health after the stressful adolescents’ transition phase than peers without psychological problems [97]. Therefore, there is more room for improvement. In addition, it has been demonstrated that girls, compared with boys, experience the transition to adolescence differently and show a greater level of psychological distress in young adolescence [11, 31]. Therefore, it is possible that gender also influences the prevention program effects [136].

Taking into account the abovementioned concerns and findings, we developed ‘Boost Camp’, which is a 2-day universal prevention program targeting basic mechanisms involved in emotion regulation for young adolescents who recently made the stressful transition from primary to secondary school. It can be expected that improving adolescents’ ER skills will significantly increase emotional wellbeing [3, 116]. The present study will test the effectiveness by means of a clustered randomized controlled trial (RCT) on primary and secondary outcomes, with long-term follow-up assessments at 3 and 6 months follow-up, taking into account important moderators.

### Goals and hypotheses

The present study has three main goals. First, this study will test the effectiveness of a new school-based universal prevention program “Boost Camp”, targeted at strengthening ER skills in the stressful first year of high school. It is hypothesized that Boost Camp will improve or maintain emotional wellbeing. More specifically, it is hypothesized that Boost Camp will lead to an *increased* use of adaptive ER, *more* positive affect, a *higher* self-esteem, and a *better* quality of life in participants in the intervention condition compared with adolescents in the control condition. Moreover, *less* negative affect, *less* use of maladaptive ER and *less* depressive and anxiety symptoms will also be seen as markers of wellbeing.

Second, this study will test the effects of the program on secondary outcomes. It is hypothesized that Boost Camp will lead to *better* peer relations, *less* negative mental health attitudes, an *increase* in academic achievements, a *smaller* amount of school absenteeism and *less* school drop-out. Third, this study will examine the moderating role of child characteristics such as EF, baseline level of psychological problems, and gender. It is hypothesized that the program will be *less* effective for young adolescents with lower levels of EF and *more* effective for young adolescents with a higher baseline level of psychological problems. Finally, due to the difference in experiencing difficulties and psychological distress within the transition phase, the program is expected to have better effects for girls than for boys.

## Methods

### Participants and procedure

Six secondary schools in the East of Flanders are contacted to participate in the study. Participants are adolescents in their first year of secondary school (7th grade, ages 11–13). The aim is to reach 300 students spread over 16 classes. We ensure the inclusion of students from different Socio Economic Status (SES) groups and various educational levels to compose a heterogeneous sample. In Belgium, there are two educational levels in secondary school. The first education level, grade A, is considered as a broad first grade in which students receive basic knowledge on technics, mathematics, or languages, with the intention to prepare them on a more specific educational program in the second grade. The second education level, grade B, is intended for students with specific needs. Both are included.

Schools that are interested are asked to participate with as many first-grade class groups as possible. If they give their consent to take part in the study, they are randomized by the researcher in a intervention or a control group. Finally, six rural schools with comparable background and meeting the same language criteria decided to participate in the study. All schools receive information letters for students and their parents, which contain detailed information about the study aims and procedure. Parents can either give or withhold their permission for including their child’s data in the study, and both parents and children are free to withdraw from the study at all times. The Boost Camp program is incorporated in the class curriculum, so all participants in the intervention condition receive the program. The advantage of incorporating the program in the class curriculum is that all adolescents participate and interact. To ensure that the intervention and the control schools place an equal emphasis on students’ mental health, teachers of all participating schools receive a workshop on care management and are invited to sign a school charter on the importance of emotional well-being of the schoolchildren.

In the intervention condition, students receive the 2-day prevention program Boost Camp followed by two extra Booster sessions. Boost Camp takes place in the beginning of the new school year, between the last week of September 2017 and the first week of October 2017. The booster sessions will be provided in the first (December 2017) and the second semester (April 2018). In the control condition, students participate in the regular school programs.

Participants will fill out online questionnaires anonymously, by using random assigned participants codes, during school hours four times throughout the first year of secondary school (Baseline, post, 3FU, 6FU). Each questionnaire session will take approximately two school hours (100 min) and will be supervised by the classroom teacher and a research assistant. Teachers and parents of participants will be asked to fill out questionnaires at two times, at the beginning (September 2017) and at the end (May 2018) of the school year. Participating classes will be rewarded with a photoshoot.

The Ethical Committee of the Faculty Psychology and Education Sciences of the Ghent University has approved the study design and data collection. Furthermore, all data collection procedures are performed in accordance with the national laws and the 1964 Declaration of Helsinki and its later amendments. During the research, any data gathered will be stored and be accessible by the primary researcher as stated in the Data Management Plan. Digital files (i.e. online questionnaires) are stored on the primary researcher’s personal computer drive, which requires a password. In addition, sensitive data (i.e. contact information) will be stored in a separate file that requires an additional password to open. Non-digital documents will be stored save in the faculty archives. Sharing a file containing personal information is out of the question.

### Design

**Fig. 1:**
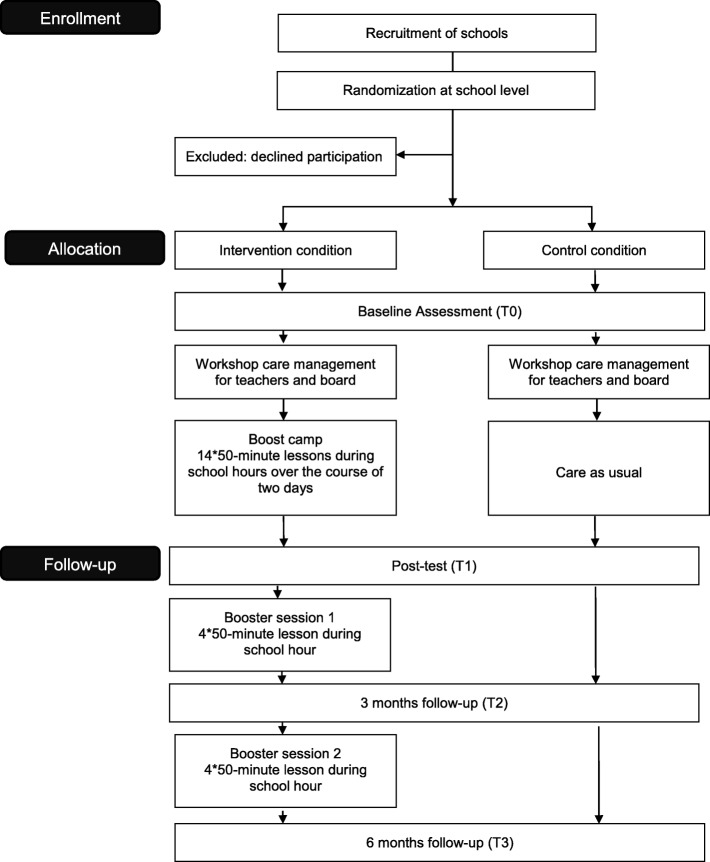
Study design

The effectiveness of Boost Camp will be tested by a clustered randomized controlled trial (cRCT) with two conditions, e.g. an intervention and a control condition (see Fig. 1). Randomization is clustered by school, which reduces the risk of contamination.

The outcomes will be measured by self-report questionnaires at the beginning of the school year (baseline: T0), immediately after Boost Camp (post -test: T1), at the beginning of the second semester (3FU, T2), and at the end of the school year (6FU, T3). Students in the control condition fill out the questionnaires in the same period as the intervention condition. Parent- and teacher-report questionnaires will be administered at baseline (T0) and at the end of the school year (T3). The trial is registered on the 13th of June 2017 at the ISRCTN Trial Register: ISRCTN68235634.

### The boost camp program

#### Theory

A well evaluated ER program is the Affect Regulation Training (ART) developed by Berking and Whitley [15]. According to this training, mastering the ART-sequence is necessary to regulate undesired affective states. The ART-sequence includes seven skills that must be acquired in a specific order, namely muscle relaxation, breathing relaxation, nonjudgmental awareness, acceptance and tolerance, compassionate self-support, analyzing emotions, and modifying emotions [13]. The ART program was designed as a stand-alone intervention to enhance adults’ adaptive ER skills by integrating techniques from various cognitive-behavioral treatment approaches, such as acceptance and commitment therapy, emotion focused therapy, mindfulness, problem solving therapies, and strength-focused interventions. ART has recently been shown to be effective in both clinical and non-clinical adult samples [12, 14–16, 26, 61].

**Fig. 2:**
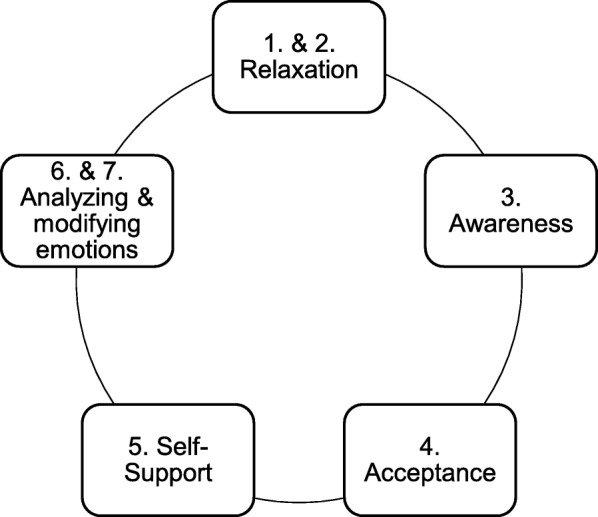
Boost Camp Modules. Adapted from “The Affect Regulation Training (ART): a transdiagnostic approach to the prevention and treatment of mental disorders,” by M. Berking and C. Lucas, 2015, *Current Opinion in Psychology*, *3*, p.66

Boost Camp is largely based on and inspired by ART. The program targets the same seven ER skills, but skills one and two are combined in one module, as well as skills six and seven (see Fig. 2). Further, important efforts were made to make the training more suited for children and adolescents. Two different pilot studies evaluated specifically step three (acceptance), four (self-support) and five (analyzing & modifying emotions) and found positive effects in young adolescents (Volkaert, Wante, Vervoort & Braet: The effect of a short emotion regulation training for young adolescents, unpublished).

The first skill, *relaxation*, refers to a behavioral technique for coping with increased levels of arousal which often coincides with negative emotional affect states [78, 89]. Relaxation techniques do not require much training and skills [83], whereby they easily can be used to facilitate adolescents’ ER efforts [98]. Decreasing the first arousal of an emotional experience is an important first step of ER as it enables an individual to accept, analyze, and modify the negative emotions. Progressive muscle relaxation [82] and breathing techniques [5] are widely used relaxation exercises in evidence-based health and children’s and adolescents’ stress regulation [21, 47, 113].

Second, *awareness* has recently been introduced as a specific ER skill that serves as a basis for learning other adaptive ER skills [15]. The aim is to focus attention on the present and observe emotions in a non-judgmental way. Hypersensitive interpretation- and appraisal processes are hereby being interrupted, which is a basic but crucial step before using cognitive resources to regulate own emotions [15]. To the best of our knowledge, awareness is not evaluated as a stand-alone technique. However, as component evidence-based programs, it has proven its merit [4, 64, 120, 137].

Third, a*cceptance* can be described as making the best of the situation. Acceptance of aversive events decreases the urge to follow inflexible patterns of avoidance, which is often known as an automatic response to escape and/or avoid aversive emotions, thoughts and body experiences [73–75]. Low levels of acceptance have been associated with different psychological problems, such as symptoms of anxiety and depressions [41, 58, 145]. Although it is stated that including a module acceptance has the potential to improve the efficacy of prevention programs [17], to date, only a couple of studies evaluated prevention programs targeting acceptance in adolescents, with mixed results. The efficacy of acceptance was examined in two school-based prevention programs among students aged 13 to 20 years. Results showed large effect sizes for the reduction in stress complaints, and medium effect sizes for the reduction in depression and anxiety scores [25, 111]. Another study evaluated the feasibility of an ACT-based prevention program among high school students aged 14 to 16 years, but found no statically significant differences between the intervention and control condition. Nevertheless, these results indicated that the intervention was feasible in a school context and suitable for the students [25].

Furthermore, *self-supporting* skills refers to satisfying basic (i.e. safety and love needs) and esteem needs (i.e. needs for self-confidence, self-esteem, self-compassion and achievement) [94], activating a strong mindset about one’s ability and personality, in order to facilitate emotional processing. A specific and well-known ER strategy that can be considered self-supporting is *distraction*, which is defined as intentionally shifting attention away from the negative emotion to an external stimulus [65]. When experiencing negative emotions, self-thoughts are often characterized by high levels of self-criticism and rejection. Consequently, additional negative feelings arise and significantly reduce the capacity to effective regulate the initial negative emotion [15]. Paying attention to an external stimulus reduces the change of the development of those additional negative feelings. Evidence for this strategy in adolescents is rather mixed, with some studies reporting positive effects (e.g. self-reported distraction is negatively associated with depressive symptoms [76]) or counter-productive outcomes (e.g. distraction is seen as cognitive avoidance, a maladaptive strategy to perceive control about a situation) [39]. Nonetheless, distraction has been shown to be effective in immediately improving adolescents’ emotional outcomes (i.e., reducing negative affect and increasing positive affect) after a negative mood induction [77, 143, 144], and combining distraction with acceptance of problem solving is thought to improve long-term effects [48, 149]. Another less-known strategy that recently is introduced as an effective ER strategy is *compassionate self-support* [15]. Self-compassion can be described as creating distance from and taking an external perspective on the suffering self and transform feelings of pain and suffer into feelings of kindness and understanding, in order to create the empathic wish to help themselves [15, 101].

The fifth and ultimate skill is to actively *analyze & modify the emotional experience* [15]. For this, two well-studied adaptive ER strategies can be used. One strategy is *cognitive reappraisal*, which can be defined as thinking in a more positive way about the affective stimuli or events [66]. Cognitive reappraisal shows good effects in decreasing an induced negative mood and increasing an induced positive mood [45, 114], and has long-lasting positive effects on emotional well-being [87, 95, 104, 124]. Training cognitive reappraisal is often a core element in various effective CBT programs for mood and anxiety disorders in both adults, adolescents and children [8, 93, 100], consistent with the observation that individuals in an anxious or depressed mood use less cognitive reappraisal compared to non-clinical samples [29, 45, 59, 107]. However, this strategy might only be effective when the level of arousal and negative emotion is mild to moderate [95]. Therefore, it can be hypothesized that cognitive reappraisal is particularly effective when it is preceded by strategies such as acceptance or distraction which actively decrease the level of arousal and emotion.

A second strategy that can be utilized to analyze and modify emotion is *problem solving,* which refers to actively modifying the stressors that induce negative affect in order to change the associated emotions. Typically, problem solving involves several steps to adequately solve a problem which may include a positive problem orientation, problem formulation, solutions generation, alternatives evaluation and process evaluation [42, 103]. Adolescents poor problem-solving skills have been associated with problem behavior [123], suicide risk [67], depressive- and anxiety symptoms [10, 115, 123, 125]. Studies that evaluate the effects of problem solving as a single and explicit ER strategy in youth are scarce. Hilt and Pollak [77] studied problem solving in a non-clinical sample of adolescents and found that the use of problem-solving did not influence induced ruminative states. However, the authors emphasize that problem-solving may have stronger effects on rumination when it is preceded by the use of distraction. Furthermore, training adolescents’ problem solving skills is often a component of cognitive-behavioral therapy (CBT) programs [43], which have been showed effective for treating various psychological disorders, such as depression [9, 20] and anxiety [140].

#### Content and structure of the boost camp program

In *the intervention condition* students are trained in five basic skills for adaptive ER (see Fig. 2).

*Day one* starts with an introduction, which aims to create a safe environment and to meet the trainers and the program. This is followed by a brief psycho-education session on different emotions and their relation with thoughts and behavior. Then, students are informed about the rational of using *relaxation- and awareness* practices and they are challenged to perform the corresponding exercises such as breathing exercises, progressive muscle relaxation, and a body scan. Next, class games are being played to learn about the different functions of emotions, which contribute to the facilitation of *accepting* and restraining intense negative emotions. Next, those functions are used to create ‘tolerance sentences’, which makes it easier to accept negative emotions and can be easily transferred to real situations. Finally, the students will be taught how to *support themselves*. First, information about the self-esteem concept and its association with mental health is given, followed by individual and group exercises to boost the students’ self-esteem. Second, students are informed about the importance of distraction and through group discussion effective distraction activities are listed. Lastly, students are challenged to create positive self-talk sentences to become a strong mindset about their ability and personality. To finish, a group activity is organized to create a frog out of paper and cardboard on which students can write their favorite sentences and take home, in order to make the transfer of the learned skills to daily life easier.

After a brief active recapitulation about the skills from day one, *day two* is fully devoted to learn how to analyze and modify an emotional experience. First, the strategy *cognitive reappraisal* is taught*.* Students learn how to be aware of their own thoughts, categorize them as helpful, unhelpful or neutral, and produce emotion and situation specific helpful thoughts through exercises and role-play. Second, students will learn and exercise the different steps to *active solve their problem*. All information, exercises and games were based on evidence based protocols [15, 18, 20, 46, 71, 131, 140] and put together in a child friendly work. Finally, t*he two boostersessions* are administered to refresh learned skills by individual- and group exercises. Both sessions takes place at school during four school hours.

#### Delivery

We planned to divide every class into two subgroups of maximum ten adolescents and with two trainers each. Groups in the B grade (i.e. children with special needs) will consist of six participants each. The training is coordinated by two main researchers (BV, LW) from Ghent University and delivered by five masters in clinical psychology and nine psychologists with at least a bachelor’s degree in clinical psychology. All trainers attend an intensive 1-day ‘train-the-trainers’ workshop and will be provided with a group leader manual, containing the different protocols for every step [142], approved by the Ethical Committee. The protocol will not be changed, at least not until the end of this data-collection. The two main researchers trained and supervised the process. They also stay on call by phone or email for support and help when needed as long as the program is active.

### Primary outcome measures

The primary outcome measures will be *change in ER* and *emotional wellbeing* immediately post-intervention, at 3 months post-intervention, and 6 months post-intervention. *ER* will be assessed by two constructs: the level of emotional awareness and the use of ER strategies*. Emotional wellbeing* will be indicated by several outcomes, namely positive and negative affect, self-esteem, quality of life, depressive- and anxiety symptoms.

#### Emotional awareness

The Dutch version of the Difficulties in Emotion Regulation Scale (DERS) is a 36-items self-report questionnaire developed to assess individuals’ identification, understanding and modulation of their own emotions [62, 102]. The DERS consists of six dimensions of emotion regulation namely Nonacceptance, Goals, Impulse, Strategies, Clarity, and Awareness. In this study, only the subscale ‘Awareness’ is administered, which reflects the tendency to percept, to attend and to acknowledge own emotions (e.g. “*I pay attention to how I feel”*) on a scale from 1 (‘almost never, 0–10%) to 5 (‘almost always, 91–100%). Higher scores indicate higher levels of awareness. The DERS demonstrated good psychometric properties for all subscales in adolescent population [102].

#### Emotion regulation

The Dutch version of the Fragebogen zur Erhebung der Emotionsregulation bei Kindern und Jugendlichen (FEEL-KJ) will be used to assess adolescents’ ER strategies [22]. The FEEL-KJ is a self-report questionnaire in which children and adolescents are asked to indicate how often they use adaptive, maladaptive, and external ER strategies in response to feelings of anger, sadness, and fear on a 5-point Likert-type scale ranging from 1 (“almost never to”) to 5 (“almost always”). The FEEL-KJ measures the use of 15 specific ER strategies. In current study, participants are asked to fill out items about 10 specific ER strategies namely *behavioral problem-solving, distraction, cognitive problem-solving, acceptance, reappraisal, revaluation, giving up, aggression, rumination, and social support.* Total scores indicates dispositions to use adaptive and *maladaptive* strategies to regulate negative emotions. The FEEL-KJ has demonstrated good psychometric properties [22, 38].

#### Positive and negative affect

The Dutch version of the Positive And Negative Affect Schedule for Children (PANAS-C) will be used to assess adolescents’ positive and negative affect [88]. Participants are asked to report in which degree, scaled from 1 (‘not at all’) to 5 (‘a lot’), they have experienced 30 positive (h*appy, energetic, proud)* and negative emotions (*vigilant, ashamed, unhappy)* in the past 2 weeks. Higher scores on the Positive Affect Score represent higher levels of positive affect, while lower scores on the Negative Affect Score represent lower levels of negative affect. The PANAS-C has shown good reliability and validity [148].

#### Self-esteem

The Perceived Competence Scale for Adolescents (CBSA) [139] is the Dutch version of the Self-Perception Profile for Adolescents (SPPA) [72] and will be used to assess self-esteem. The 35-items self-report questionnaire aims to measures six specific domains of self-worth, namely scholastic competence, social acceptance, athletic competence, physical appearance, behavioral conduct, close friendships, and global self-worth. In current study, only the last subscale will be used (e.g. ‘*some youngster are often disappointed in themselves, but other youngster are almost never disappointed in themselves).* Higher scores indicate a higher global self-worth. Research about validity and reliability is convincing [139].

*Quality of life* will be measured by the Dutch version of the KIDSCREEN-10, which is a short measure for assessing adolescents’ well-being and health-related quality of life (HRQoL) The KIDSCREEN-10 consist of 10 items (e.g.*, ‘Do you have felt full of energy?’)*, which are rated on a five-point scale ranging from 1 to 5 [112]. Higher scores represent a higher subjective health related quality of life. The KIDSCREEN-10 showed good psychometric properties [54].

#### Depressive symptoms

Depressive symptoms will be measured with the Dutch versions of the Child Depression Inventory (CDI) [86, 138], and the shortened version of the Center for Epidemiological Studies Depression scale (CES-D) [109]. The CDI consist of 27 items, which can be answered by choosing one out of three statements that best describes his or her feelings in the past 2 weeks. For example*, ‘I feel like crying everyday’, I often want to cry’, ‘I sometimes feel like crying’.* A score can be assigned to each statement, ranging from 0 to 2 [86]. The total score gives an indication of the severity of the self-reported depressive symptoms. The CDI has demonstrated satisfactory reliability and validity for measuring depressive symptoms [86, 138], but is also known as a less sensitive and reliable instrument to assess low to mild depressive symptoms [90]. Therefore, the 9-items version of the CES-D is also used. Adolescents are asked to indicate how much they have experienced an item in the past week, ranged from ‘never’ = 0, to ‘always’ = 3. For example*, ‘I was bothered by things that usually don’t bother me’* or *‘I felt fearful’.* The higher the total score, the more depressive symptoms are reported. The CES-D has shown acceptable reliability and validity in adolescent populations [110].

#### Anxiety symptoms

Anxiety symptoms will be measured with the Dutch version of the State–Trait Anxiety Inventory for Children (STAI-C) [7, 132]. The STAI-C initially consists of two subscales, but in current study, only the subscale on ‘state anxiety’ will be administered. The subscale consists of 20 items (e.g. *‘I feel happy now’)*, followed by three answer possibilities ranging from “hardly ever” to “often”. Higher scores indicate greater anxiety. High reliability and satisfactory validity are showed [133].

### Secondary outcome measures

Secondary outcomes include Dutch versions of instruments assessing social behavior, such as a*ttitudes about psychological stigmatization, relationships with peers and bullying,* and academic performance, such as *school achievement*, *school absence,* and *drop-out*.

#### Attitudes about psychological stigmatization

Adolescents’ attitudes about psychological stigmatization will be assessed by an adolescent-friendly version of the Stigma Scale for Receiving Psychological Help (SSRPH) [106]. Adolescents have to indicate whether they agree on five statements about stigmatization (e.g. *‘Going to the psychologist is a sign of personal weakness’* or ‘*Other people think it’s weird to visit a psychologist’)* on a scale range from 1 (‘Strongly disagree’) to 5 (‘Strongly agree’). A higher score indicate a higher degree of public stigma. Beginning evidence for the validity and reliability of the SSRPH in adolescent population is shown [106].

#### Relationship with peers and bullying

The Dutch version of the Olweus Bully/Victim Questionnaire (OBVQ) will be used to assess self-reported relationships with peers and bullying behavior [91, 105]. Adolescents are asked to report on their relationships at school (e.g. *‘How many good friends do you have in the classroom?’*) and to score items on being bullied in the present and the past (e.g. *‘How often have other students bullied you this school year?*’) [91]. Higher scores on part one represents better relationships with peers. Higher scores on part two represents more bullying experience. The scale has a good variability and reliability [128].

#### Academic performance

S*chool achievement* will be assessed by teachers, adolescents and school orientation. Teachers are asked to fill out the Academic Performance Rating Scale (APRS) [50], a rating scale for assessing perceptions from teachers about students’ academic competencies. This 19-items questionnaire assesses a range of specific achievement parameters, like work performance (e.g. *‘Estimate the percentage of math work completed, compared to classmates’*), academic success (e.g. *‘How quickly does this child learn new material?’)*, behavior control (e.g. *‘How often is the child able to pay attention without you prompting him/her?’)* and attention to assignments (e.g. *‘How frequently does the student accurately follow teacher instructions and/or class discussion during small-group (e.g., reading group) instruction*?). The APRS has shown high reliability and good validity [50]. Next to the teacher-report version of the APRS, adolescents are also asked to rate how they score on courses such as mathematics and languages compared with their classmates on a 5-point Likert scale, ranging from 0 = ‘really bad’ to 5 = ‘really good’. Finally, school orientation attests will be requested at group level at the end of the school year to assess how many students passed their first year of secondary school. At the end of the school year the total number of *absences* and the students that *drop-out* through the year will be requested.

### Moderators

Current study will include possible moderators, like initial psychological problems, EF, and gender.

*Initial psychological problems* will be assessed by the Dutch version of the Child Behavior Checklist [1, 141], which is a parent-report questionnaire on adolescents’ internalizing and externalizing symptoms. Parents are asked to score 113 problem items which were presented (0), sometimes presented (1), or very often present (2) in the past 6 months, and can be divided into eight subscales, namely. Anxiety/depressive, withdrawal/depressive, somatic complaints, social problems, thought problems, attention problems, antisocial behavior, and aggressive behavior. The scores on these subscales can be summed up to yield total scores on internalizing problems, externalizing problems, and total problem level [1, 141]. The CBCL is a well validated and reliable questionnaire [1, 141].

*EF* will be assessed by the Dutch parent version of the Behavior Rating Inventory of Executive Functioning (BRIEF) [60, 126]. The BRIEF parent version aims to assess adolescents’ everyday EF by asking parents to score their child’s behavior on a 3-point Likert scale, ranging from ‘never’ to ‘frequently’. The questionnaire consists of 86 items that can be divided into eight subscales, namely *inhibition, shifting, emotional control, initiation, working memory, planning/organizing, organizing materials,* and *monitoring.* In current study, the global executive score will be used. Higher scores indicate weaker executive functioning. The BRIEF has revealed a good reliability and validity [126].

Finally, information on socio-demographic factors including *gender* will also be obtained from both child and parents.

### Feasibility indices

Fidelity and treatment integrity of the trainers will be assessed to estimate the feasibility of the program. Each trainer will be asked to indicate to what extent the program has been followed, the goals that have been reached, and how well they were able to guide and help the adolescents during the Boost Camp program. Further, trainers and adolescents will fill out an evaluation form about their experience of the Boost camp program with regard to the group atmosphere, adolescents’ motivation and program comprehensibility. Lastly, the program components will be taped and scored by independent coders in order to check feasibility. An overview of measurements and time points can be found in Table 1.

**Table 1:** An overview of measurements and time points

Concept	Measurement (items)	Measurement in time points			
		T0	T1	T2	T3
Primary outcome measure					
ER					
Emotional awareness	DERS	x	x	x	x
ER strategies	FEEL-KJ	x		x	x
Emotional wellbeing					
Positive and negative affect	PANAS-C	x	x	x	x
Self-esteem	CBSA	x	x	x	x
Quality of Life	HRQoL	x		x	x
Depressive symptoms	CDI	x	x	x	x
	CES-D	x	x	x	x
Anxiety symptoms	STAI-C	x	x	x	x
Secondary outcome measure					
Social behavior					
Attitudes about stigmatization	SSRPH	x	x	x	x
Relationship with peers and bullying	OBVQ	x	x	x	x
Academic Performance					
School achievement	ARSP	x			x
	Self-report question	x			x
	School Orientation Attest				x
School drop-out	School data				x
School absence	School data				x
Moderators					
Initial psychological problems	CBCL	x			x
Executive functioning	BRIEF	x			x
Gender	Self-report question	x			

Note: FEEL-KJ = the Fragebogen zur Erhebung der Emotionsregulation bei Kindern und Jugendlichen [22], DERS = Difficulties in Emotion Regulation Scale [62], PANAS = Positive And Negative Affect Schedule for Children [88], CDI = Children’s Depression Inventory [85], CES-D = Center for Epidemiological Studies Depression Scale [109], STAI-C = State-Trate Anxiety Inventory for Children [7], CBSA = Competence Belief Scale for Adolescents [139], HRQoL = KIDSCREEN-10 well-being and health-related quality of life [112], CBCL = Child Behavior CheckList [141], OBVQ = Olweus Bully/Victim Questionnaire [91], SSRPH = Stigma Scale for Receiving Psychological Help [106], ARSP = Academic Performance Rating Scale [50], BRIEF = Behavior Rating Inventory for Executive Function [60] T0 = Baseline assessment, T1 = Post-test, T2 = 3 months follow-up, T3 = 6 months follow-up

### Data analysis

Power analyses were calculated to determine the required sample size for detecting a clinically significant difference between the intervention and control group at 6 months follow-up on one primary outcome (FEEL-KJ). With a significance level of α = 0.05 and a power level of β = 0.90, a total sample of 86 N_Non-cluster_ participants is needed to detect a significant difference. However, the clustering of participants within schools has to be considered and therefore, the design effect is calculated as 1 + (m-1)*ICC, with m referring to the number of participants per cluster [57]. Based on prior research determining the ICC values for adolescent health outcomes [121], an Intraclass Correlation Coefficient (ICC) of 0.02 was used. The estimated average size of the classes participating in current study is counted on 20 students per class, resulting in a design effect of 1,38. Moreover, as the intervention requires a high time investment, an 3:2 ratio in favor of the control group will be chosen at randomization. Following the recommendations of Gao et al. [57], the final calculation of the total sample of the control group was multiplied by the design effect with the randomization ratio and the N_Non-Cluster,_ resulting in a total sample size of 241 students, 145 for the control group and 97 for the intervention group. Taking into account declined participation and loss to follow-up, a drop-out rate of 20% is estimated. Finally, this resulted in a required sample size of 291 students, 174 in the control group and 117 in the intervention group, clustered in at least 16 classes.

Assumptions will be checked by examining descriptive statistics and missing variables will be examined. When data are missing completely at random, maximum likelihood estimation will be used to estimate the missing values. When data are not missing completely at random, generalized estimating equation analyses will be used. Possible baseline differences in both groups will be assessed using simple t-tests or chi-square tests, and variables that show baseline differences will be entered as covariates of no interest in all models. To assess the difference between the intervention and control condition in the change in the use of adaptive ER skills and in emotional wellbeing during 6 months following the program, a mixed model will be used. More specifically, a mixed model with fixed effects for condition (intervention or control), time (immediately after intervention, 2 months post FU, and 6 months post FU), and their interaction, adjusted for baseline will be used. Moderators will be added one by one. To account for the nested structure of students within classes, and classes within schools, a random intercept for schools and a random intercept for classes nested within schools will be utilized. To account for the temporal correlation of measurements within a student, an unstructured residual covariance will be assumed.

## Discussion

Boost Camp is an innovative universal school-based program addressing ER ability in young adolescents, based on Berking’s model and ART [15]. In the current study, the effectiveness of Boost Camp in boosting well-being after making the stressful transition from primary to secondary school will be tested by a RCT. In addition, the study will examine whether child characteristics, such as initial psychological problems, executive functioning, or gender moderates the program’s effectiveness. It is hypothesized that, post-intervention adolescents in the Boost Camp condition will display more adaptive ER skills and a better emotional wellbeing, indicated by more positive affect, a higher self-esteem, a better quality of life, less negative affect, less use of maladaptive ER, and less depressive and anxiety symptoms, up until 6 months follow-up, compared with the adolescents in the control condition. Furthermore, it is expected that gender, initial psychological problems and executive functioning moderate the intervention effect on emotional wellbeing.

### Strengths and limitations

The study has numerous important strengths. First of all, we use a stringent design including an attention control group, randomization, 6 months follow-up, and adhering to CONSORT guidelines, to evaluate the effect of the intervention in a real life school setting. Both control and intervention schools receives a workshop on the importance of students’ emotional wellbeing in the school context for teachers and principals, in order to control the intervention school’s attention for wellbeing [56]. Furthermore, the randomization at school-level is most feasible, increases the generalizability of the results and minimizes contamination. Second, in addition to the effect of the Boost Camp program on primary outcomes, also the effects on secondary outcomes such as academic achievement, relationships with peers, and attitudes about psychological problems are assessed. Noteworthy, academic achievement is measured through multiple informants and multiple-assessment, towards a more nuanced understanding and consistent with the multi-method, multi-informed approach advocated in developmental psychopathology research. Third, the Boost Camp program is based on well-evaluated theory and practices. Theory is based on the positively evaluated ART and content relies on evidence based protocols for children and adolescents [13, 18, 20, 71, 130, 140]. Moreover, transdiagnostic programs that aim to promote social and emotional skills may enhance the efficacy and cost-effectiveness of prevention programs and be critical in adolescents’ health prevention [36, 49]. Various therapeutic approaches and clinical programs have already incorporated some modules targeting ER skills [13, 20, 21, 130]. The inclusion of these modules in addition to care as usual seems to have positive effects on adolescents’ and adults’ wellbeing in various clinical contexts [127]. Fourth, the study will also evaluate the implementation process. Train-the-trainer is provided by qualified trainers; trainers will be asked to evaluate the program and independent raters will be asked to check for fidelity and integrity.

However, some limitations of the present study can be identified as well. First of all, the randomization at school-level increases the risk of different participants characteristics, like social economic status, which in turn decreases the chance to create a perfectly matched and equivalent control group and increases the risk of selection bias [80]. Furthermore, there is no active control group used, because up until now, there is no other existing Dutch school-based ER prevention program to which Boost Camp could be compared. Second, emotional wellbeing is only assessed by self-report measures which includes possible response biases through social desirability or a lack of introspection [6]. Finally, participants and trainers were not blind to the allocated intervention, through which it is possible that adolescents in the intervention condition try harder to be aware and effective regulating their emotions, which is known as the Hawthorne Effect [80]. However, educational research trials often lack blinding as a design feature due to the high visibility of the interventions. Moreover, other evaluation methods cannot improve this because they also will not operate blinding [80].

### Implication for practice

If the results of this study demonstrate that Boost Camp is effective in improving ER ability, maintaining or increasing emotional wellbeing, and decreasing symptoms of psychopathology, it will have valuable and beneficial effects for adolescents, their broader environment like teachers and family, and society. Besides, the risk that these adolescents become adults in need decreases. Furthermore, the transition from primary to secondary school can be considered as a stressful life-event and as a result of the extensive pressure on young adolescents to perform well on behavior and academic work, the need for prevention programs focusing on strengthen resilience factors among young adolescents is high. If the results of this study demonstrate that Boost camp is effective, it can easily be implemented through organizing Train-the-Trainer workshops, whereby an expert trains, supervises and monitors school psychologists. In addition, both the moderators as well as the trial will provide knowledge about the effectiveness of the program for high-risk adolescents, which will allows us to develop future programs to be adapted to the needs of all adolescents.
